# Management approach for recurrent spontaneous pneumothorax in consecutive pregnancies based on clinical and radiographic findings

**DOI:** 10.1186/1749-8090-1-35

**Published:** 2006-10-19

**Authors:** Eric Scott Sills, Henry M Meinecke, George R Dixson, Alan M Johnson

**Affiliations:** 1 Department of Obstetrics, Gynecology and Reproductive Research, Murphy Medical Center, Murphy, North Carolina USA; 2 Department of Surgery, Murphy Medical Center. Murphy, North Carolina USA; 3 Department of Radiology, Murphy Medical Center. Murphy, North Carolina USA; 4 Asheville Cardiovascular & Thoracic Surgeons, P.A., Asheville, North Carolina USA

## Abstract

**Objective:**

To describe management and clinical features observed in a patient's seven spontaneous pneumothoraces that developed during two consecutive pregnancies involving both hemithoraces.

**Materials and methods:**

A 21 year old former smoker developed three spontaneous left pneumothoraces in the index pregnancy, having already experienced four right pneumothorax events in a prior pregnancy at age 19.

**Results:**

Chest tubes were required in several (but not all) hospitalizations during these two pregnancies. Following her fourth right pneumothorax, thoracoscopic excision of right apical lung blebs and mechanical pleurodesis was performed. The series of left pneumothoraces culminated in mini-thoracotomy and thoracoscopically directed mechanical pleurodesis. For both pregnancies unassisted vaginal delivery was performed with no adverse perinatal sequelae. With the exception of multiple pneumothoraces, there were no additional pregnancy complications.

**Conclusion:**

Spontaneous pneumothorax in pregnancy is believed to be a rare phenomenon, yet the exact incidence is unknown. Here we present the first known case of multiple spontaneous pneumothoraces in two consecutive pregnancies involving both hemithoraces. Clinical management coordinated with obstetrics and surgical teams facilitated a satisfactory outcome for both pregnancies. The diagnosis of pneumothorax should be contemplated in any pregnant patient with dyspnea and chest pain, followed by radiographic confirmation.

## Background

Spontaneous pneumothorax in pregnancy is generally regarded as an unusual disorder, with only approximately 50 cases having been reported in the world literature. Here we describe diagnosis and treatment for a patient who experienced multiple pneumothoraces developing in two consecutive pregnancies.

## Case report

### Previous pregnancies

Three years before initial presentation, a 19 year-old female experienced the first of seven spontaneous pneumothorax events. By that time, three first trimester miscarriages had occurred (etiology unknown) and she had already undergone an uncomplicated term vaginal delivery of a healthy male infant. The patient did not use alcohol but had a four pack-year smoking history; she had stopped smoking 10d before her first pneumothorax. In the sixth week of her fifth pregnancy, she "felt sharp right-sided chest pain" radiating posteriorly accompanied by right arm paraesthesia and dyspnea. This developed while driving her car home. There was no history of trauma, coughing or sneezing episodes. She was brought to the emergency room where she was admitted with the diagnosis of right pneumothorax and a chest tube was placed. Good re-expansion was noted on chest x-ray and the patient was discharged home 2d later.

Approximately two weeks later, a second pneumothorax occurred, this time following a prolonged "vomiting spell" secondary to hyperemesis of pregnancy. Evaluation in the emergency room identified a recurrent right pneumothorax. The patient's pulmonary symptoms were less pronounced and it was not considered large enough on chest x-ray to warrant treatment with a chest tube.

A third small right pneumothorax developed at the 10^th ^gestational week that responded well to conservative management and, again, no chest tube was required.

However at ~19 weeks' gestation, the patient sustained a fourth pneumothorax with symptoms similar to that of the first. Chest x-ray revealed approximately 50% collapse of the right lung; she agreed to referral for definitive surgical management. Thoracoscopic excision of right apical lung blebs and mechanical pleurodesis proceeded without complication. The remainder of pregnancy was unremarkable and she was delivered (vaginally) of a viable female infant at term. Of note, the infant was diagnosed with postaxial polydactyly of both hands and one foot. Speech therapy identified "limited vocabulary for ag" and developmental delay was diagnosed. A normal karyotype was confirmed in the infant.

### Final pregnancy (index presentation)

At age 21 the patient presented for prenatal care of her sixth pregnancy. She had resumed smoking following her penultimate delivery, but had discontinued cigarettes seven months before this conception. Based on her miscarriage history, additional testing was undertaken during antenatal screening and the patient was found to have protein S deficiency and be heterozygous both for MTHFR and prothrombin G20210A genes. Perinatal consultation determined that anticoagulation therapy was unnecessary based on these findings. Other prenatal laboratory tests and cervical cytology were normal. At approximately five weeks' gestation, the patient experienced "sharp left chest pain" and a spontaneous left pneumothorax was diagnosed by chest radiograph. Her previous four pneumothoraces involving the contralateral (right) hemithorax were noted. With supportive care her condition improved and lung re-expansion was achieved without chest tube placement. The patient was discharged home after three days.

For her sixth pneumothorax, she was readmitted to hospital one week later with similar symptoms and left pneumothorax was again identified on chest x-ray. Seven days of supportive care was followed by clinical improvement and radiographically confirmed resolution of the pneumothorax; the patient was discharged from hospital in good condition.

Thirteen days later, the patient described another episode of "extreme left-sided chest pain". Left pneumothorax was found on chest x-ray (50% collapse of the left lung). A 20 French chest tube was placed under intravenous sedation (midazolam HCl 2 mg) and lung re-expansion to <5% pneumothorax was achieved. Her hospital course during this seventh pneumothorax was unremarkable except for a mild intercurrent pneumonia (T_max_38.2°C) that responded well to intravenous antibiotics. The patient was discharged home in good condition 19 days later.

At 16 weeks' gestation the patient was admitted to hospital for mini-thoracotomy and thoracoscopically directed mechanical pleurodesis. Apical bleb disease was not prominent, and no obvious source of leak was identified. Recovery was uneventful and complete re-expansion of the lung was achieved.

**Figure 1:**
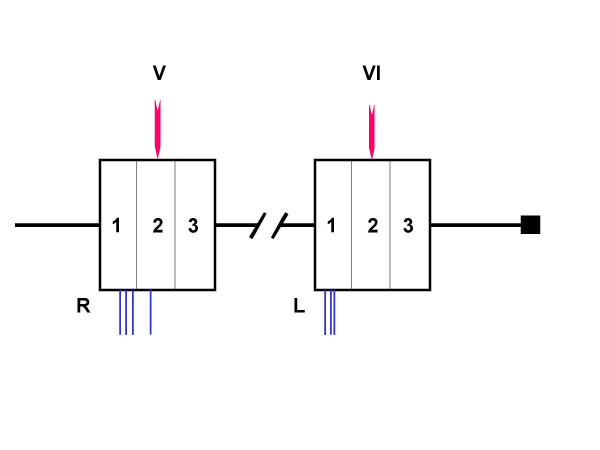
Relative frequency of spontaneous pneumothoraces is marked by blue lines below rectangles depicting the two serial pregnancies organized by trimester. Red arrows indicate timing of thorascopy (pregnancy V) or mini-thoracotomy/thorascopy (pregnancy VI). R = right, and L = left pneumothorax. Black box at terminus represents bilateral fallopian tubal sterilization performed 10 weeks following the sixth pregnancy.

She experienced no further chest pain and the remainder of her pregnancy was uncomplicated. At 34 5/7 weeks' gestation the patient underwent a spontaneous vaginal delivery of a viable male infant (weight 2336 g, Apgars 9/9). Given her tendency to develop pneumothoraces in pregnancy, the patient requested permanent surgical sterilization and laparoscopic bilateral tubal ligation was performed 10 weeks later. The progression of recurrent pneumothoraces and their treatment during the two pregnancies is depicted in Figure 1. Significant chest x-ray findings observed during the two pregnancies are summarized in Table 1.

**Table 1:** Summary of selected chest radiograph findings during two consecutive pregnancies with multiple spontaneous pneumothoraces

Hospitalization		% pneumothorax on CXR
R	1	30*
	2	<10
	3	<10
	4	50**
L	5	
	6	<10
	7	50**

*Notes: *R spontaneous pneumothoraces occurred in pregnancy #1–4, L spontaneous pneumothoraces occurred in pregnancy #5–7. (*) indicate hospitalizations associated with chest tube placement. (**) denotes surgical procedure to correct pneumothorax. CXR = chest x-ray.

## Discussion

Spontaneous pneumothorax is a pathologic condition of extrapulmonary air within the chest accompanied by lung collapse without trauma to the lung or chest wall. It is encountered more frequently in males than in females and is particularly uncommon in pregnancy. While only approximately 50 such cases have been reported to date, the precise incidence is not known. Some investigators have observed that typical pneumothorax symptoms such as chest pain and dyspnea are often attributed to paroxysmal tachycardia, neuralgia, or asthma exacerbation – thus contributing to underreporting of spontaneous pneumothorax in the literature [1]. Although previous authors have described spontaneous pneumothorax in pregnancy [2-6], including one case with four recurrent spontaneous pneumothorax episodes occurring before the end of the second trimester [7], ours is the first report to describe recurrent spontaneous pneumothoraces in two consecutive pregnancies involving both hemithoraces.

Rupture of small bullae has been suggested as one cause for primary spontaneous pneumothorax, while secondary spontaneous pneumothorax develops in the setting of pre-existing lung disease such as chronic obstructive pulmonary disease (COPD), asthma, tuberculosis, pneumonia, cystic fibrosis, lung malignancies, and certain types of interstitial lung disease. Differential diagnoses include Langerhans histiocytosis, tuberous sclerosis, and lymphangioleiomyomatosis. Prompt, accurate diagnosis is essential as sudden death (secondary to tension pneumothorax) may occur before chest tube placement, and respiratory collapse can happen hours after tube insertion even with careful monitoring.

While chest radiographs in pregnancy may present concerns for patients, a standard two-view film is associated with only 7 × 10^-5 ^rad radiation exposure. In contrast, the generally accepted cumulative dose of ionizing radiation that may be safely permitted in pregnancy is 5 rad [8]. For our patient, no chest CT studies were performed.

The presence of pulmonary blebs (sometimes evident from the early neonatal period) could predispose an individual to spontaneous pneumothorax in adulthood, but since our patient was herself delivered at home by a midwife no chest x-rays were obtained. Although speculative, if such a lesion existed in our patient it is plausible that the increased minute ventilation, tidal volume, and respiratory rate with diminished functional residual capacity of pregnancy [9] might have contributed to the pregnancy-associated pneumothoraces identified here.

As illustrated in this report, treatment of spontaneous pneumothorax is based on evacuation of air from the pleural space permitting the lung to re-expand. Hospitalization and chest tube placement has been a standard treatment for spontaneous pneumothorax for many years. For our patient, lung re-expansion in some instances required several days with a chest tube, while only non-invasive supportive care was needed in others.

Although surgery may be indicated for recurrent pneumothorax episodes, specific criteria for operative intervention are lacking. Certainly the intercurrrent pregnancies characteristic of this case presented an additional complication. Nevertheless, preventive measures should include smoking cessation, avoidance of rapid or drastic change in ambient pressure such as high altitudes, scuba diving, or flying in unpressurized aircraft. As this case illustrates, recurrent pneumothorax warrants consideration in any pregnant patient with acute chest pain, dyspnea or history of prior pneumothorax.

## Competing interests

The author(s) declare that they have no competing interests.

## Authors' contributions

ESS, HMM, GRD and AMJ all contributed to and approved the manuscript.
